# Esthetic outcome for implant therapy of a maxillary lateral incisor using prefabricated titanium and customized zirconia abutments: 4‐year clinical reports

**DOI:** 10.1002/ccr3.4983

**Published:** 2021-10-21

**Authors:** Gerardo Guzman‐Perez, Carlos Alberto Jurado, Carla Araciel Rincon‐Reyna, Saad Alresayes, Ysidora Torrealba, Abdulrahman Alshabib, Chin‐Chuan Fu, Akimasa Tsujimoto

**Affiliations:** ^1^ CEMRO Periodontics Residency Program Morelia Mexico; ^2^ Prosthodontist Texas Tech University Health Sciences Center El Paso Woody L. Hunt School of Dental Medicine El Paso Texas USA; ^3^ Private Practice Irapuato Mexico; ^4^ Prosthetic Dental Sciences Department King Saud University College of Dentistry Riyadh Saudi Arabia; ^5^ Department of Restorative Dentistry University of Alberta Faculty of Medicine and Dentistry Edmonton Canada; ^6^ Department of Restorative Dentistry King Saud University College of Dentistry Riyadh Saudi Arabia; ^7^ Department of Restorative Sciences University of Alabama at Birmingham School of Dentistry Birmingham Alabama USA; ^8^ Department of Operative Dentistry University of Iowa School of Dentistry Iowa City Iowa USA

**Keywords:** dentistry

## Abstract

The reported clinical scenarios presented two patients, one managed with a prefabricated abutment and the other with a customized abutment, and both patients were pleased with the outcome. However, from a professional viewpoint, the esthetic outcome using the custom zirconia abutment was superior to that using the prefabricated titanium abutment.

## Graphical abstract

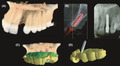

## 1 INTRODUCTION

Implant therapy has proven to be an effective form of rehabilitation for partially or fully edentulous patients, with success rates exceeding 90%, and thus, the therapy has become popular with patients and a routine task for clinicians.^1^ On the contrary, although implant therapy has a high success rate and is long‐lasting, current patient and clinician demands are moving beyond the simple survival of the restoration to consider how well it harmonizes with the surrounding teeth and soft tissue and how long it functions as a natural‐looking and beautiful tooth.^2^ These esthetic demands have become paramount, especially for patients seeking restorations in the esthetic zone.^3^

Anterior single implants represent a complex clinical situation for both surgeons and restorative clinicians due to the need to meet specific constraints set by the adjacent natural teeth.^4^ In particular, cases in which a single anterior tooth has been lost in an accident are held to be especially difficult. The handling of the soft tissue surrounding the implant needs to create the illusion of a natural tooth and be in harmony with adjacent teeth.^5^ In general, any implant‐supported crown placed in the anterior zone presents a challenge for the clinician because it will be displayed in the patient's smile. According to a novel classification in implant therapy, any implant in the anterior maxilla region needs to be classified as either advanced or complex.^6^

Prefabricated titanium abutments have been used for a long time and treated by some authors as the reference standard for implant restorations due to the metal's reported stability during clinical studies.^7^ The easy assembly of their components makes prefabricated abutments a good option. In addition, the cost of prefabricated abutments and the final restorations is much lower than that of customized abutments and restorations for custom abutments. However, prefabricated abutments have a cylindrical shape that very often needs to be modified in order to achieve a tooth‐like shape in the implant prosthesis, and the soft tissue needs to be guided up from the gingiva to follow the contour needed for the full restoration, regardless of the shape of the abutment.^8^ In addition, prefabricated abutments have a predetermined crown margin height that may not follow the patient's gingival position and architecture.^8^ This may create a discrepancy between the position of a prefabricated abutment and the gingival tissue, which may compromise the removal of cement, in cases where the final restoration is cemented and may increase the risk of peri‐implantitis.^9^ Moreover, the silver color of titanium stock abutments may lead to dark discoloration in the soft tissue surrounding the implant.^10^

Custom abutments, which are made of either titanium, gold, or zirconia, were developed to overcome the limitations of the prefabricated abutments.^8^ Custom abutments can be fabricated to achieve an optimal outline, emergence profile, gingival margin, and space needed for the crown. This makes it easier to contour the soft tissue to match the final restoration. The esthetic outcome is superior for zirconia abutments than for titanium or gold abutments because zirconia abutments are either white or match the tooth color, which avoids issues of grayish discoloration in the peri‐implant tissue.^10^ In addition, zirconia abutments have shown similarly fracture rates to metal abutments.^11^ Cases have been reported that used customized zirconia abutments to achieve esthetic and functional restorations, and these benefits have been emphasized. However, there have been no reports comparing the postoperative development of restorations performed in similar cases by the same operators, using both prefabricated titanium abutments and customized abutments.

Therefore, this case report aims to describe a comparison of the esthetic outcome of implant therapy of a maxillary lateral incisor using prefabricated titanium and customized zirconia abutments.

## 2 CLINICAL REPORTS

### 2.1 Case one

**FIGURE 1:**
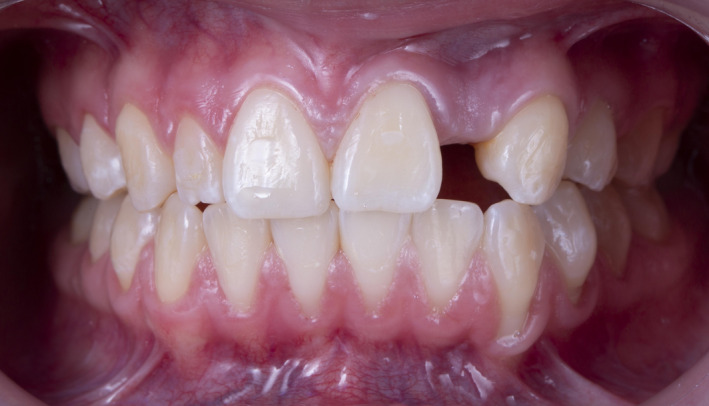
Initial clinical situation frontal view

A 25‐year‐old female patient presented to the clinic with the chief complaint of “I need an implant” (Figure 1). The patient was referred by a general dentist and stated she lost her #10 tooth due to an accident 5 years ago. The patient was using a removable prosthesis but disliked it and was seeking a fixed dental prosthesis. Medical history was reviewed, and no illnesses and diseases were found and patient was classified as ASA class I as a normal healthy patient. Cone‐beam computed tomography (CBCT; Instrumentarium OP300, Renew Digital LLC) was used to evaluate the area three‐dimensionally and assist in planning the ideal implant position (Software 4.5.9 Blue Sky Plan). The scan identified the presence of bone deficiency and the need for a bone graft on the buccal area of the site (tooth #10).

The need for a simultaneous bone graft during the implant therapy was explained to the patient, who agreed to the procedure after being informed of her options. A Kirkland type flap was performed, and a single implant with a diameter of 3.2 mm and length of 11.5 mm (ETIII Implant, Hiossen Implant) was placed. A bone graft (A‐Oss, Osstem Implant Co) was placed, and a membrane (Creos Allo Protect Membrane, Nobel Biocare) with three screws (Titanium Bone Tacks, Salvin Dental Specialties Inc) secured the bone graft into position. The soft tissue flap was repositioned, and vertical and horizontal mattress suturing techniques provided closure (Cytoplast Non‐Absorbable PTFE Sutures, Osteogenics Biomedical). A polymethylmethacrylate (Jet Tooth Shade, Lang Dental Manufacturing Co) Adhesive Interim prosthesis (E‐max CAD‐CAM milled block—lithium disilicate glass‐ceramic—Ivoclar Vivadent) was cemented onto the lingual surface of tooth #9 and cantilevered on #10. The provisional prosthesis was kept completely out of occlusion during the healing time.

**FIGURE 2:**
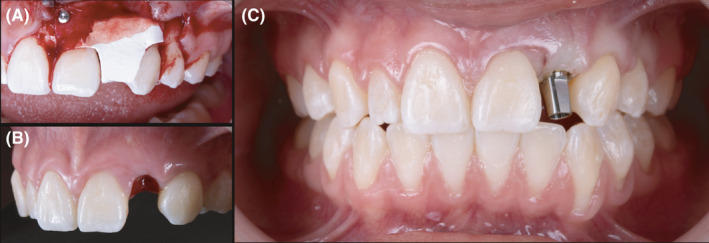
(A) Bone grafting procedures. (B) Soft tissue contoured. (C) Prefabricated custom abutment

After 2 months, another provisional restoration (Temp‐Bond, Kerr Corporation) was fabricated directly onto the implant to help contour the soft tissue. Provisional restoration contours were modified every 2 weeks for 2 months, at which time the soft tissue provided an ideal gingival architecture. An impression abutment (Mini Hex, Hiossen Implant) was placed, and a final impression was made with polyvinyl silane in heavy and light body consistency (Extrude VPS, Kerr Corporation). A prefabricated abutment (FreeForm ST Abutment, Hiossen implant) was evaluated in the mouth, and contours were evaluated. (Figure 2).

**FIGURE 3:**
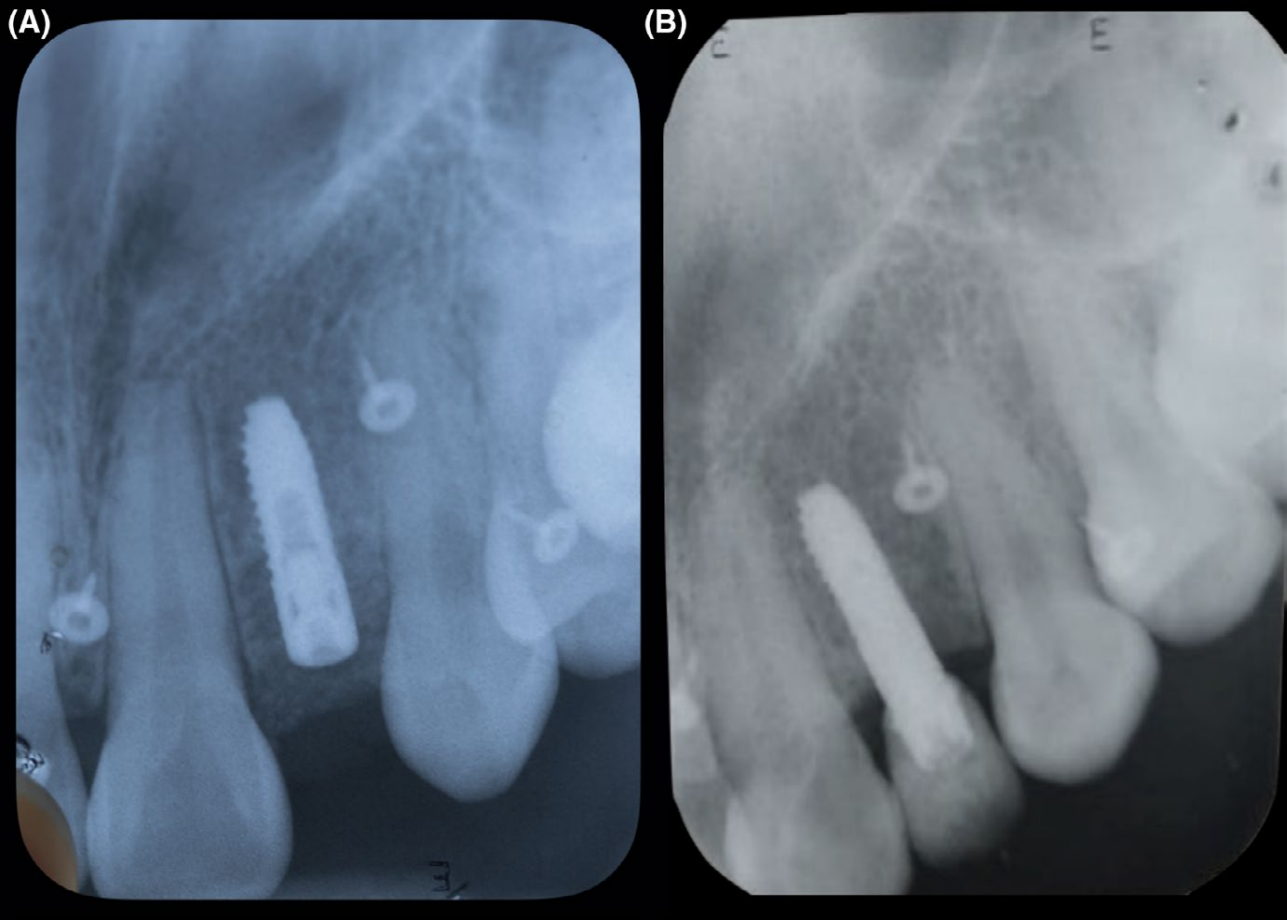
(A) Radiograph: initial placement. (B) Radiograph: 4 years follow‐up

**FIGURE 4:**
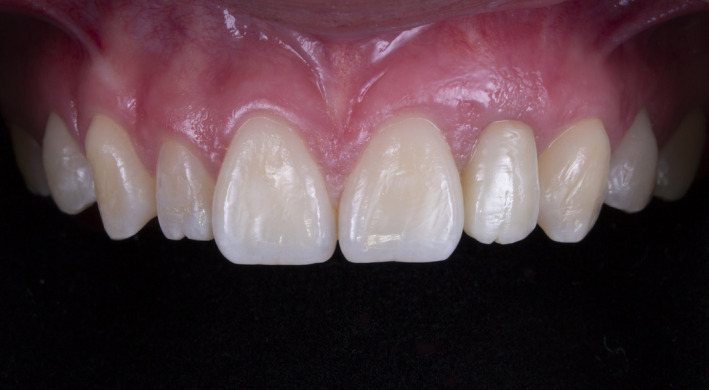
Four years follow‐up frontal view

Finally, a porcelain fused to zirconia restoration crown was fabricated. The stock abutment was placed back in position, and the manufacturer's recommended torque was completed. Occlusion was evaluated and adjusted as needed to ensure the crown had minimal contact on MIP and no contact on protrusive movement. A retraction cord #2 was placed around the abutment to prevent cement displacement. The final crown was cemented on to the abutment using self‐adhesive resin cement (Rely X Unicem, 3 M Company) on the abutment. The patient was satisfied with the final results and she received a night guard to protect her teeth and the dental restoration. At the 4‐year follow‐up, the patient was still pleased with the result. (Figures 3 and 4).

### 2.2 Case two

**FIGURE 5:**
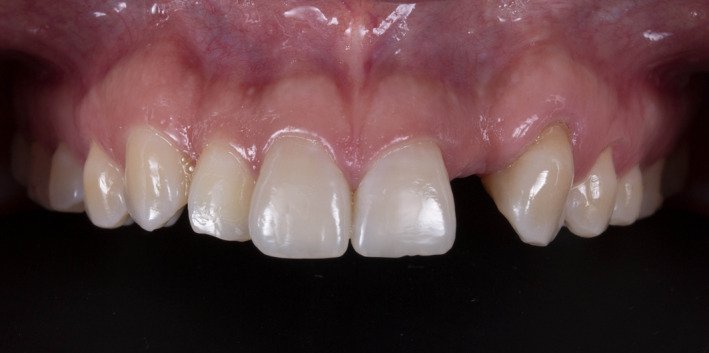
Initial clinical situation frontal view

A 28‐year‐old female patient presented to the clinic with the chief complaint of “I lost a front tooth, and I want to replace it” (Figure 5). Patient presented with a missing tooth #10 and stated that the tooth had been endodontically treated and restored with a porcelain fused to metal crown several years earlier. However, 2 years ago, the same tooth #10 was removed due to mobility and secondary caries around the restoration. The patient was not wearing any prosthesis. Patient presented with no history of major medical illness and taking no medication so she was classified as an ASA class 1. The patient was offered different treatment plan options, including a removable prosthesis, an implant, and a three‐unit tooth‐supported fixed restoration. The patient elected to have implant therapy.

A diagnostic wax‐up was made to evaluate the dimensions and position of the final restoration. A cone‐beam computed tomogram CBCT (CS 8100 3D, Carestream Dental LLC) of the patient was taken, and a three‐dimensional evaluation was completed (4.5.9 Blue Sky Plan). The assessment found sufficient bone in the area that provided the ideal position for the implant and followed the curvature of the adjacent teeth. The patient was also offered the option of having soft tissue graft on the facial gingival recession of tooth #11, which the patient accepted. The diagnostic wax‐up was superimposed on the CBCT to fabricate a surgical guide for the ideal position of the implant (Photon, Anycubic).

An implant with a 3.0 mm diameter and a length of 11.5 mm was placed (ETIII Implant, Hiossen Implant) on site #10. Soft tissue auto‐grafts taken from the palate were placed on the facial surface of tooth #11 using non‐resorbable sutures (Cytoplast Non‐Absorbable PTFE Sutures, Osteogenics Biomedical). The non‐resorbable sutures, along with resin composite, were placed on the facial surface of tooth #11 to help hold the tissue in position (Filtek Supreme, 3 M Company). Next, a screw‐retained provisional restoration, made using polymethylmethacrylate, for tooth #10 was placed in position. The contour of the provisional restoration was modified every other week for 2 months to construct an ideal gingival architecture. Once the tissue obtained the desired shape, a final impression was made.

**FIGURE 6:**
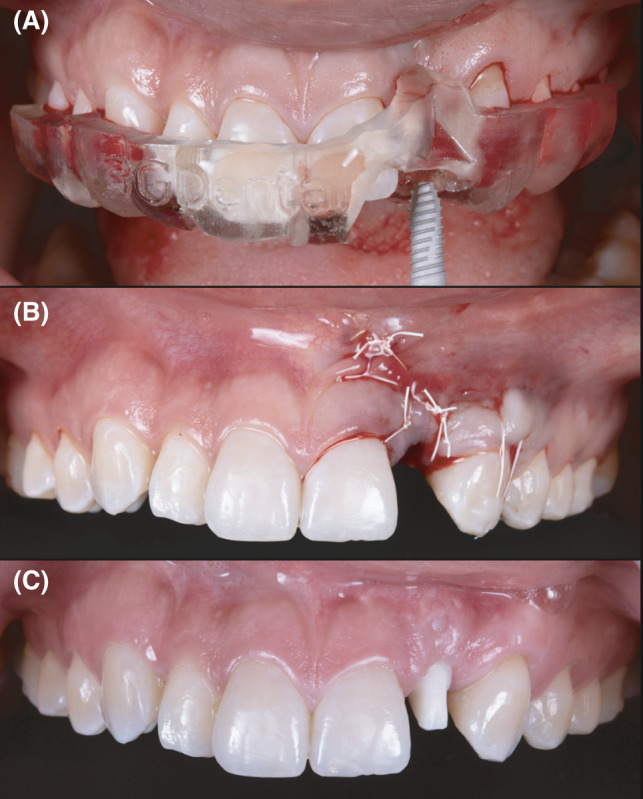
(A) Implant placement. (B) Grafting and suturing. (C) Customized zirconia with titanium base abutment

**FIGURE 7:**
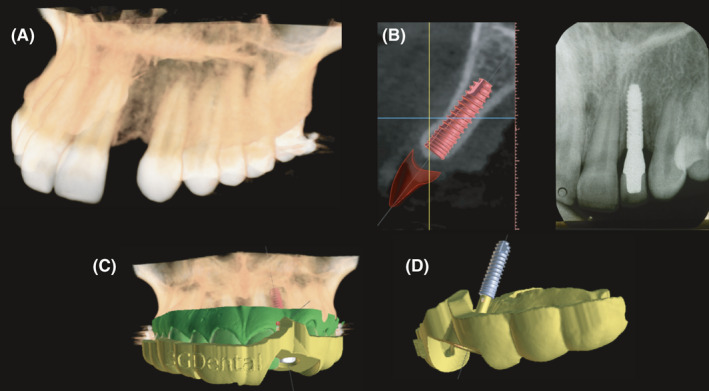
(A and B) Implant planning. (C and D) Guide planning. (E) Four years follow‐up

**FIGURE 8:**
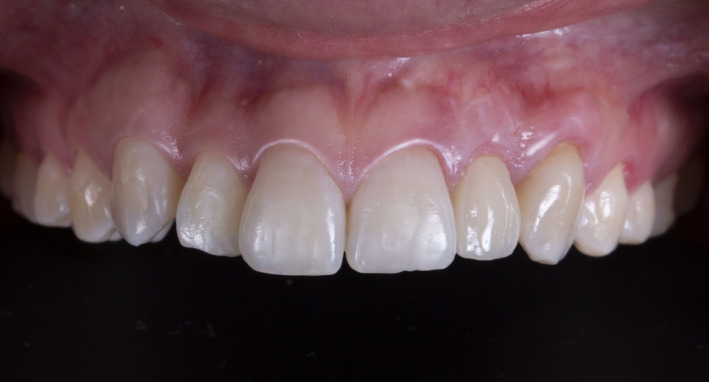
Four years follow‐up frontal view

A zirconia with titanium base abutment and porcelain fused to zirconia crown were fabricated. The custom titanium base abutment was placed in the mouth, and contours were evaluated. (Figure 6) A retraction cord was placed around the abutment to prevent any subgingival cement displacement. Then, the final porcelain fused to zirconia crown was cemented (Rely X Unicem, 3 M Company) onto the custom abutment. Occlusion was evaluated and adjusted as needed. The patient was happy with the final result, and a night guard was provided to protect her teeth and the restoration. The patient was still pleased with the outcome at the 4‐year follow‐up. (Figures 7 and 8).

## 3 DISCUSSION

The ideal three‐dimensional placement of the implant results in optimal support and stability for the surrounding hard and soft tissues.^12^ The position of the implant directs the emergence profile of the crown which replaces the missing tooth. In most cases, the tentative final restoration is used to guide implant placement because it represents the desired form and position.^13^ In both these clinical cases, a cone‐beam computed tomogram (CBCT) was taken to evaluate the bone three‐dimensionally, and they were considered with a diagnostic wax‐up model. Superimposing the wax‐up scan on the CBCT helped develop a surgical guide and fabrication plan to achieve the desired position. It was possible to place the implant in the ideal position through the use of the surgical guide.

For proper decision‐making, the clinician must predict how different gingival phenotypes could react to implant‐related therapy.^13^ The thickness of the buccal bone is one of the most important clinical parameters in achieving predictable esthetic outcomes for anterior dental implants.^14^ Another factor to take into consideration is the presence of keratinized gingiva, which plays a vital role in the long‐term maintenance and survival of dental implants.^14^ Keratinized gingiva is considered to be beneficial for the stability of peri‐implant soft tissue because it forms an antimicrobial barrier. In the first clinical scenario, a buccal deficiency was noted, and the grafting procedure was carried out simultaneously with the implant placement. In the second case, only soft tissue grafting was provided to address the gingival recession of tooth #11. Due to these grafting procedures, the optimal outcomes were achieved.

At the 4‐year follow‐up for both clinical cases presented here, the soft tissue levels reminded stable without any reduction. This can be attributed to the development of these techniques. Implant placement and bone grafting techniques have evolved and made surgical results more predictable for clinicians. It is clear both from earlier reports and from these cases that if these two procedures are performed appropriately, patient satisfaction can be obtained. As a result, three‐dimensional implant planning, implant position, and surgical management for soft tissue are more important than the type of abutment.

However, from a professional view point, the esthetic outcome was much better for the implant therapy using a customized zirconia abutment than for that using a prefabricated titanium abutment due to the darkened soft tissue in the latter case. It goes without saying that this is due to the influence of the abutment color. Although the problem is at a level that does not bother the patient, these cases confirmed that, considering the color of the material, the use of zirconia offers a superior prognosis for the restoration. Further, the restorative procedures involved in implant therapy in the esthetic zone present challenges in soft tissue management beyond those of color.^15^ An ideal implant restoration can be achieved by mimicking the natural contours of teeth and gingiva. Flattened tissue needs to be recontoured during the provisional restoration, and papilla should be allowed to close gingival embrasures. Comparing the two cases, it is clear that suitable soft tissue contours were achieved much more quickly with the customized abutment. The patient with the prefabricated abutment had a provisional restoration period of 4 months, during which the shape of the provisional restorations was adjusted several times in the buccal and interproximal areas until the desired tissue contour was obtained. On the contrary, in the case of the customized zirconia abutment, it took just 2 months, half the time, to achieve the gingival embrasure. This can be attributed to the fact that a zirconia custom abutment can be shaped to match the ideal gingival contour, and the biocompatibility of zirconia makes it possible to apply a shaping force to the soft tissue immediately after placing the abutment. This shows that, if the patient's budget permits, a customized zirconia abutment will secure a higher quality result.

## 4 CONCLUSION

In these two cases, both prefabricated titanium and custom zirconia abutments were successfully used to achieve the desired esthetic outcomes over 4 years. Properly planned and precisely placed implants and gingival management can be achieved using either prefabricated titanium or custom zirconia abutments. However, the esthetic outcome in implant therapy for a maxillary lateral incisor using a custom zirconia abutment was better and faster than that using a prefabricated titanium abutment.

## CONFLICT OF INTEREST

The authors declare that there is no conflict of interest regarding the publication of this article.

## AUTHOR CONTRIBUTIONS

GGP and CAJ contributed to concepts and manuscript editing. CARR and YT contributed to design. SA and CCF contributed to definition of intellectual content. AA and AT contributed to manuscript editing. AT and CCF contributed to literature search and manuscript review.

## ETHICAL APPROVAL

The patients described were fully informed on the method and the purpose of the case report. Written consent to participate and for publication was obtained by the patients and is available upon request.

## CONSENT

All authors have confirmed during submission that patients' consents have been signed and collected in accordance with the journal's patient consent policy.

## Data Availability

The data that support the findings of this study are available from the corresponding author upon reasonable request.

## References

[1] Jurado CA , Tsujimoto A , Guzman LG , et al. Implant therapy with monolithic translucent zirconia restorations in the esthetic zone. Gen Dent. 2020;68(1):46‐49. 31859662

[2] Buser D , Chappuis V , Belser UC , Chen S . Implant placement post extraction in esthetic single tooth sites: when immediate, when early, when late? Periodontol 2000. 2017;73(1):84‐102. 28000278 10.1111/prd.12170

[3] Jurado CA , Watanabe H , Villalobos‐Tinoco J , Ureta Valenzuela H , Guzman PG , Tsujimoto A . A conservative approach to ceramic veneers: a case report. Oper Dent. 2020;45(3):229‐234. 31860390 10.2341/19-051-T

[4] Stefanini M , Felice P , Mazzotti C , Mounssif I , Marzadori M , Zucchelli G . Esthetic evaluation and patient‐centered outcomes in single‐tooth implant rehabilitation in the esthetic area. Periodontol 2000. 2018;77(1):150‐164. 29493024 10.1111/prd.12215

[5] Arunyanak SP , Pollini A , Ntounis A , Morton D . Clinician assessments and patient perspectives of single‐tooth implant restorations in the esthetic zone of the maxilla: a systematic review. J Prosthet Dent. 2017;118(1):10‐17. 28385430 10.1016/j.prosdent.2016.10.036

[6] Higginbottom F , Belser U , Jones JD , Keith SE . Prosthetic management of implants in the esthetic zone. Int J Oral Maxillofac Implants. 2004;19:62‐72. 15635946

[7] Angkaew C , Serichetaphongse P , Krisdapong S , Dart MM , Pimkhaokham A . Oral health‐related quality of life and esthetic outcome in single anterior maxillary implants. Clin Oral Implants Res. 2017;28(9):1089‐1096. 27412520 10.1111/clr.12922

[8] Zarauz C , Pitta J , Pradies G , Sailer I . Clinical recommendations for implant abutment selection for single‐implant reconstructions: customized vs standardized ceramic and metallic solutions s restorative dent. Int J Periodontics Restorative Dent. 2020;40(1):31‐37. 31815970 10.11607/prd.3913

[9] Mahn DH . Implant abutment and restoration design and risk factors for peri‐implant disease. Compend Contin Educ Dent. 2019;40(9):572‐576. 31573216

[10] Naveau A , Rignon‐Bret C , Wulfman C . Zirconia abutments in the anterior region: a systematic review of mechanical and esthetic outcomes. J Prosthet Dent. 2019;121(5):775‐781. 30617036 10.1016/j.prosdent.2018.08.005

[11] Coray R , Zeltner M , Özcan M . Fracture strength of implant abutments after fatigue testing: a systematic review and a meta‐analysis. J Mech Behav Biomed Mater. 2016;62:333‐346. 27239815 10.1016/j.jmbbm.2016.05.011

[12] De Lemos AB , Kahn S , Rodrigues WJ . Influence of periodontal biotype on the presence of interdental papillae. Gent Dent. 2013;61:20‐24. 24064158

[13] Martin WC , Pollini A , Morton D . The influence of restorative procedures on esthetic outcomes in implant dentistry: a systematic review. Int J Oral Maxillofac Implants. 2014;29:142‐154. 24660196 10.11607/jomi.2014suppl.g3.1

[14] Chen ST , Buser D . Esthetic outcomes following immediate and early implant placement in the anterior maxilla–a systematic review. Int J Oral Maxillofac Implants. 2014;29:186‐215. 24660198 10.11607/jomi.2014suppl.g3.3

[15] Testori T , Weinstein T , Scutellà F , Wang HL , Zucchelli G . Implant placement in the esthetic area: criteria for positioning single and multiple implants. Periodontol 2000. 2018;77(1):176‐196. 29484714 10.1111/prd.12211

